# Cryoballoon vs. laser balloon ablation for atrial fibrillation: a meta-analysis

**DOI:** 10.3389/fcvm.2023.1278635

**Published:** 2023-12-18

**Authors:** Xiaochi Sun, Shenyu Zhao, Simin Yu, Kaijun Cui

**Affiliations:** ^1^ Department of Cardiology, West China Hospital, Sichuan University, Chengdu, Sichuan, China; ^2^ West China Medical School, Sichuan University, Chengdu, Sichuan, China

**Keywords:** atrial fibrillation, catheter ablation, cryoballoon, laser balloon, meta-analysis

## Abstract

**Background:**

Cryoballoon ablation (CBA) and laser balloon ablation (LBA) are two innovative ways for the treatment of atrial fibrillation (AF). This study aimed to evaluate the efficacy and safety of cryoballoon ablation and laser balloon ablation in patients with AF.

**Methods:**

We searched Pubmed, Embase, Ovid, Web of Science and other databases for comparative trials comparing CB and LB ablation in the treatment of AF, from establishment of database to August, 2023.

**Results:**

A total of 13 studies and 3,582 patients were included (CBA, *n* = 2,308; LBA, *n* = 1,274). There was no difference between CBA and LBA in acute PVI rate per vein, 12-months recurrence rate of AF, 12-months recurrence rate of atrial arrhythmia, occurrence rate of pericardial tamponade, occurrence rate of inguinal complications. LBA presented a lower acute PVI rate per patients (CBA 97.0% vs. LBA 93.4%, RR = 1.04, 95%CI: 1.01–1.07). Transient nerve palsy was more likely to occur after CBA (CBA 2.7% vs. LBA 0.7%, RR = 4.25, 95%CI: 2.06–8.76). However, the occurrence of persistent nerve palsy between CBA and LBA groups were similar (CB 1.4% vs. LB 1.0%, RR = 1.09, 95%CI: 0.55–2.14). In terms of procedural duration, the procedural time of CBA was shorter than that of LBA (WMD = −26.58, 95%CI: −36.71–16.46).

**Conclusions:**

Compared with LBA, CBA had a shorter procedural duration. There was a higher incidence of transient but not persistent phrenic nerve palsy after CBA.

**Systematic Review Registration:**

https://www.crd.york.ac.uk/prospero/display_record.php?RecordID=272607 Identifier (CRD42021272607).

## 1. Background

Atrial fibrillation (AF) is the foremost common arrhythmia in grown-ups. According to a epidemiological study, the lifetime risk of AF in people over 40 years of age was 26.0% for men and 23.0% for women (1). Moreover, AF may lead to several serious complications such as heart failure and stroke, ultimately leading to a 1.5–2 fold increase in all-cause mortality (2).

Pulmonary vein isolation (PVI) through catheter ablation has become a major method for the treatment of atrial fibrillation. Cryoballoon ablation (CBA) is one of the most widely applied ablation methods. The cryoballoon uses refrigerant to rapidly reduce the temperature in the fine tube, which causes cryogenic damage to adjacent pulmonary vein tissue, achieving the purpose of PVI. CBA provides numerous advantages over the most often used radiofrequency catheter ablation, including a shorter learning curve, shorter procedure duration, and less periprocedural problems (3, 4).

Recently, laser balloon ablation (LBA), which takes advantage of laser energy, has become a new method for AF intervention. During LBA, the operator can gain intracardiac vision through an endoscope mounted at the tip of the catheter, thus leading to a shorter learning curve than radiofrequency catheter ablation. One study showed that even in the early period of the learning curve, LBA was as safe and effective as radiofrequency catheter ablation in the treatment of AF (5).

Several studies directly comparing the efficacy and safety of CBA and LBA in the treatment of AF showed mixed results (6–18). This study conducted a meta-analysis to evaluate efficacy and safety of CBA and LBA in the treatment of AF in order to provide evidence for clinical intervention of AF.

## 2. Methods

### 2.1. Search strategy

This review was performed according to the Preferred Reporting Items for Systematic Reviews and Meta-Analyses (PRISMA) statement (19). From inception until August 2023, scientific databases such as Pubmed, Embase, Ovid, and Web of Science were searched for papers comparing CB vs. LB ablation treatment for AF. The following key words and MeSH terms were used in the searches: “cryoballoon”, “cryoablation”, “laser balloon”, “atrial fibrillation” “pulmonary vein isolation” and “trial”. The search strategy for Pubmed was (“Cryoballoon” OR “Cryoablation”) AND (“Laser balloon” OR “Laserballoon”) AND (“Atrial fibrillation” OR “AF”) AND (“pulmonary vein isolation” OR “PVI”).

### 2.2. Inclusion and exclusion criteria

Two investigators independently screened the title and abstracts of searched literature. Full text of the literature included in the first step would be assessed. Disagreements would be solved by a third investigator.

Inclusion criteria were as follows: (i) randomized or non-randomized studies comparing the efficacy and safety regarding CB vs. LB ablation with or without abstract; (ii) studies enrolling more than 20 patients who meet the diagnostic criteria in the AHA/ACC/HRS guidelines (20) for the management of patients with atrial fibrillation; (iii) studies including any of the first outcome variables, such as the acute PVI rate, the 12-months recurrence rate of atrial fibrillation and atrial arrhythmia and the occurrence of periprocedural complications.

Exclusion criteria were as follows: reviews, meta-analysis, case-reports, non-comparative studies, studies without outcomes of interest and overlapping reports.

### 2.3. Quality accessment

Quality assessment was conducted through the method recommended by Cochrane using the Cochrane Collaboration risk of bias tool (21). Three investigators independently assessed study quality.

### 2.4. Definitions of outcomes

The primary efficacy outcome was rate of the acute PVI and freedom from atrial fibrillation and any atrial tachyarrhythmia after 12 months without anti-arrhythmic agents administration. Recurrence during a 90-day blanking period is not considered as a true recurrence. The primary safety outcome was the occurrence of complications, including deaths, atrioesophageal fistula, cardiac tamponade, embolic events, phrenic nerve palsy (PNP) and groin complications. Second outcome was procedure time.

### 2.5. Statistical analysis

Statistical analysis was performed using Revman 5.3 and Stata 16.0. To assess the heterogeneity of the included studies, Cochran's *Q* test and the *I*^2^ statistic were used. If *P* ≥ 0.1 and *I*^2 ^≤ 50%, it indicated that studies were relatively homogeneous and fixed effect model would be applied. If *P* < 0.1 or *I*^2^ > 50%, and no obvious clinical heterogeneity was observed, the random effect model would be used for combination. For subsequent analysis, continuous data was transformed into weighted mean difference (WMD) with 95% confidence intervals (CI), while binary data was turned into risk ratios (RRs) with 95% CI. Publication bias was tested using funnel plots and Egger test.

## 3. Results

### 3.1. Search results

**Figure 1:**
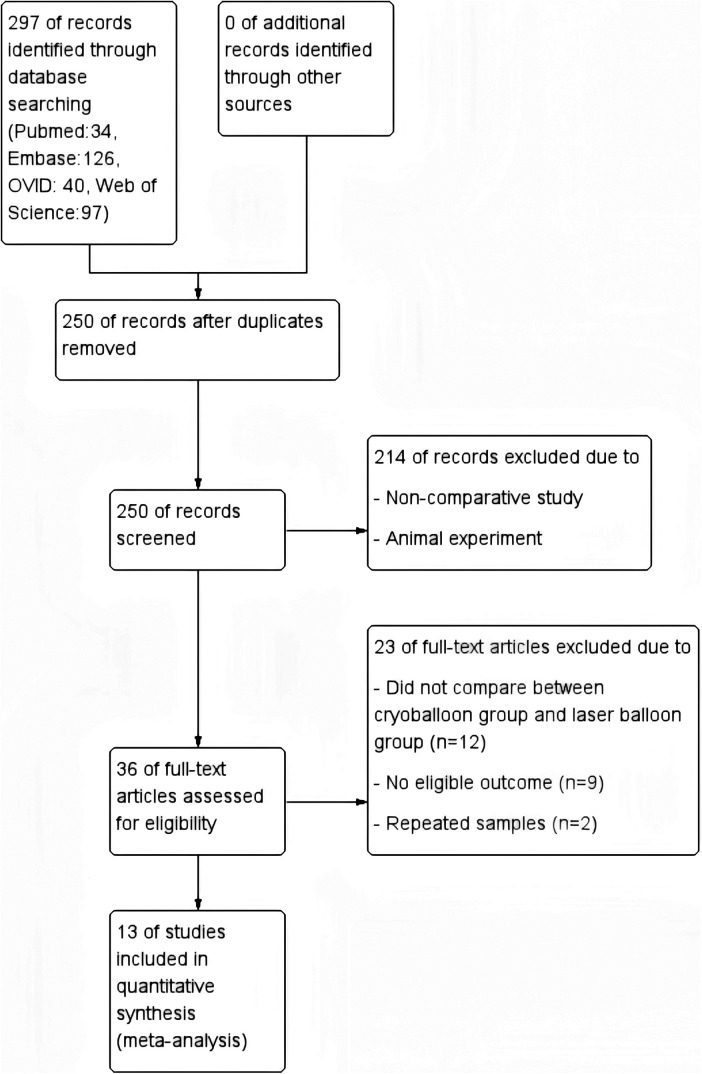
Literature search and selection process.

A total of 292 publications were initially retrieved from Pubmed, Embase, Ovid and Web of Science databases. After screening on the basis of the inclusion criteria, a total of 13 literatures were eventually included for analysis. All the studies included have obtained ethical approvals. Figure 1 revealed the literature search and selection process, and the quality assessment of the included literatures is shown in Supplementary Figure S1.

### 3.2. Basic characteristics of included studies

Characteristics of included studies are summarized in Supplementary Table S1. In all the studies, antigulation was sufficiently implemented. Activated clotting time of patients was maintained between 300 and 400 s or international normalized ratio was maintained between 2 and 3. Second-generation CB (Arctic Front Advance, Medtronic Inc.) was applied in most studies, and in all the studies visually guided laser ablation catheter was applied.

A total of 13 literatures, including 3,637 patients (2,343 CBA, 1,294 LBA) in total and 1,149 patiens with primary efficacy outcome (588 CBA, 561 LBA). In total, acute PVI rates were reported in 9 studies (8–16). 12-months recurrence rates were reported in 6 studies (6–8, 11, 13, 18). Complication rates were reported in 11 studies (6–11, 13, 14, 16–18). Procedural time was reported in 11 studies (7–16). 5 studies included both paroxysmal atrial fibrillation (PAF) and persistent atrial fibrillation (PersAF) patients (7, 9, 11, 14, 17), while 8 studies included only PAF patients (6, 8, 10, 12, 13, 15, 16, 18). Main characteristics of studies were summarized in Table 1 and Supplementary Table S1.

**Table 1:** Characteristics of included studies.

Author (publish year)	Research type	Following-up duration	Inclusion criteria	*N* (CBA)	*N* (LBA)	Male (%, CBA/LBA)	LB Ablation	CB Ablation
Bordignon et al. (10) 2013	RCT	12 months	Paroxysmal AF refractory to AAD	140	70	70.0%/61.4%	First-generation laser balloon	Second-generation 28 mm cryoballoon
Casella et al. (6) 2014	RCT	12 months	Paroxysmal AF refractory to AAD	35	20	65.7%/85.0%	First-generation laser balloon	First-generation 23-mm or 28-mm balloon (Arctic Front™, Medtronic) and second-generation ion cryoballoon
Chun et al. (11) 2021	Prospective	12 months	Paroxysmal (<7 days) or persistent (>7 days and <1 year) AF refractory to AAD (PAF: *n* = 50; PersAF: *n* = 50)	100	100	58.0%/54.0%	First-generation and second-generation laser balloon (first *n* = 32 and second generation *n* = 68)	Second-generation CB (SC, Achieve, 20 mm, Medtronic, Minneapolis, MN)
Conti et al. (12) 2015	RCT	12 months	Paroxysmal AF	50	40	NR	First-generation laser balloon	Second-generation 28-mm CB (Arctic Front Advance, Medtronic Inc.)
Huang et al. (13) 2021	RCT	NR	Paroxysmal AF	115	115	NR	Third-generation Laserballoon (Heartlight X3; LB3)	Third-generation cryoballoon (Arctic Front Advance-Short Tip; CB3)
Kumar et al. (14) 2014	Retrospective	12 months	Paroxysmal and persistent AF refractory to AAD (PAF: *n* = 53; PersAF: *n* = 7)	40	20	72.5%/75.0%	First-generation laser balloon (HeartLight™, CardioFocus)	Second-generation CB (Arctic Front™, Medtronic; CB, Medtronic, Minneapolis, MN, USA)
Yano et al. (18) 2021	Prospective	12 months	Paroxysmal AF refractory to AAD	55	56	58.2%/57.1%	First-generation laser balloon	Second-generation CB over an inner-lumen circumferential mapping catheter (Achieve™, Medtronic).
Perrotta et al. (7) 2017	RCT	12 months	Paroxysmal and persistent AF refractory to AAD (PAF: *n* = 32; PersAF: *n* = 8)	20	20	65.0%/80.0%	First-generation laser balloon (HeartLight™, CardioFocus)	Second-generation 28-mm balloon (Arctic Front Advance™, Medtronic)
Schmidt et al. (15) 2013	Retrospective	NR	Paroxysmal AF refractory to AAD	33	33	NR	First-generation laser balloon	First-generation 28-mm balloon (Arctic Front™, Medtronic)
Stockigt et al. (16) 2016	Retrospective	12 months	Persistent or longstanding AF refractory to AAD	35	35	56.2%/56.5%	First-generation laser balloon (HeartLight™, CardioFocus)	Second-generation 28-mm CB (Arctic Front Advance, Medtronic Inc.)
Tohoku et al. (17) 2020	Prospective	12 months	Paroxysmal and persistent AF refractory to AAD (PAF: *n* = 1,523; PersAF: *n* = 910)	1,720	713	66.0%/60.0%	NR	NR
Tsyganov et al. (8) 2015	Prospective	NR	Paroxysmal AF refractory to AAD	50	50	70.0%/65.9%	First-generation laser balloon	Second generation cryoballoon (Arctic Front Advance™, Medtronic, MN, US)
Wissner et al. (9) 2014	Prospective	12 months	Paroxysmal and short-standing persistent AF refractory to AAD (PAF: *n* = 50; PersAF: *n* = 14)	20	22	70.0%/66.0%	First-generation laser balloon	Second-generation 28-mm balloon (Arctic Front Advance™, Medtronic)

### 3.3. Meta-analysis results

#### 3.3.1. Acute PVI rate

**Figure 2:**
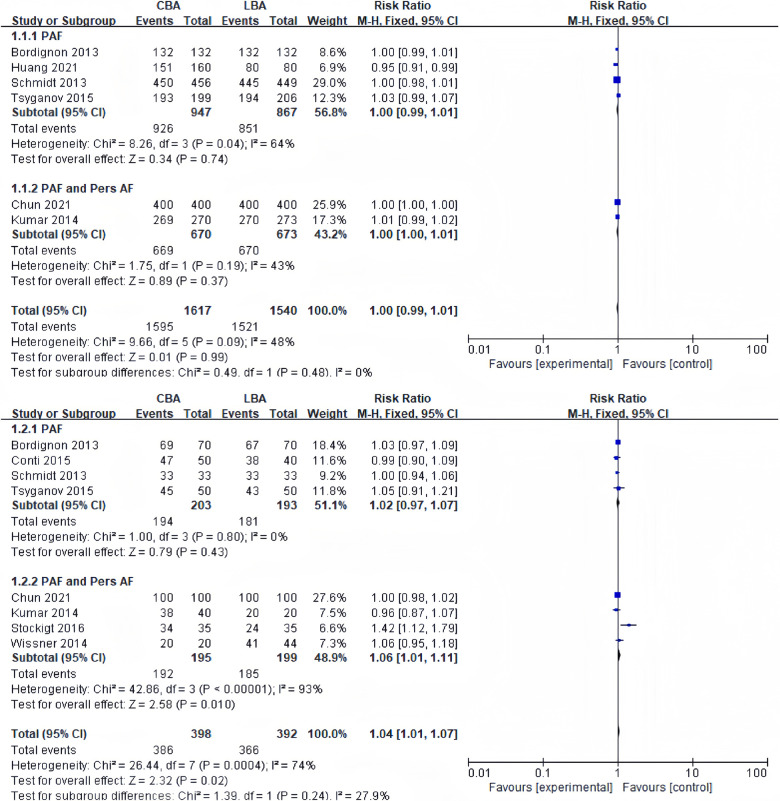
(**A**) Forest plot demonstrating acute PVI rate per vein in CBA compared to LBA; (**B**) forest plot demonstrating acute PVI rate per patient in CBA compared to LBA.

9 of the 13 studies included in the meta-analysis recorded acute PVI rates. 6 studies reported acute PVI rates per vein. Heterogeneity tests revealed no significant heterogeneity between studies (*I*^2 ^= 48%, *p* = 0.09), and a fixed-effect model was applied for synthesis. The results revealed that acute PVI rate per vein in the CBA group was 98.7% (1,595 veins/1,617 veins), and that in the LBA group was 98.8% (1,521 veins/1,540 veins) with no significant difference (RR = 1.00, 95%CI: 0.99–1.01) (Figure 2A).

8 studies reported acute PVI rates per patient. Heterogeneity tests revealed significant heterogeneity between studies (*I*^2 ^= 74%, *p* = 0.33), and randomized-effect models were applied for synthesis. The results revealed that acute PVI rate per patient in the CBA group was 97.0% (386 patients/398 patients), and that in the LBA group was 93.4% (366 patients/392 patients), and the difference was statistically significant (RR = 1.04, 95%CI: 1.01–1.07) (Figure 2B). Sensitivity analysis revealed that the difference became no longer statistically significant after excluding the study by Stockigt (RR = 1.01, 95%CI: 0.98–1.04) (16).

Considering acute PVI rate, the subgroup analysis showed no significant difference between CBA and LBA in both subgroup (subgroup containing only PAF patients and subgroup containing both PAF and PersAF patients).

#### 3.3.2. 12-months heart arrhythmia recurrence (AF and atrial tachyarrhythmia) rate

**Figure 3:**
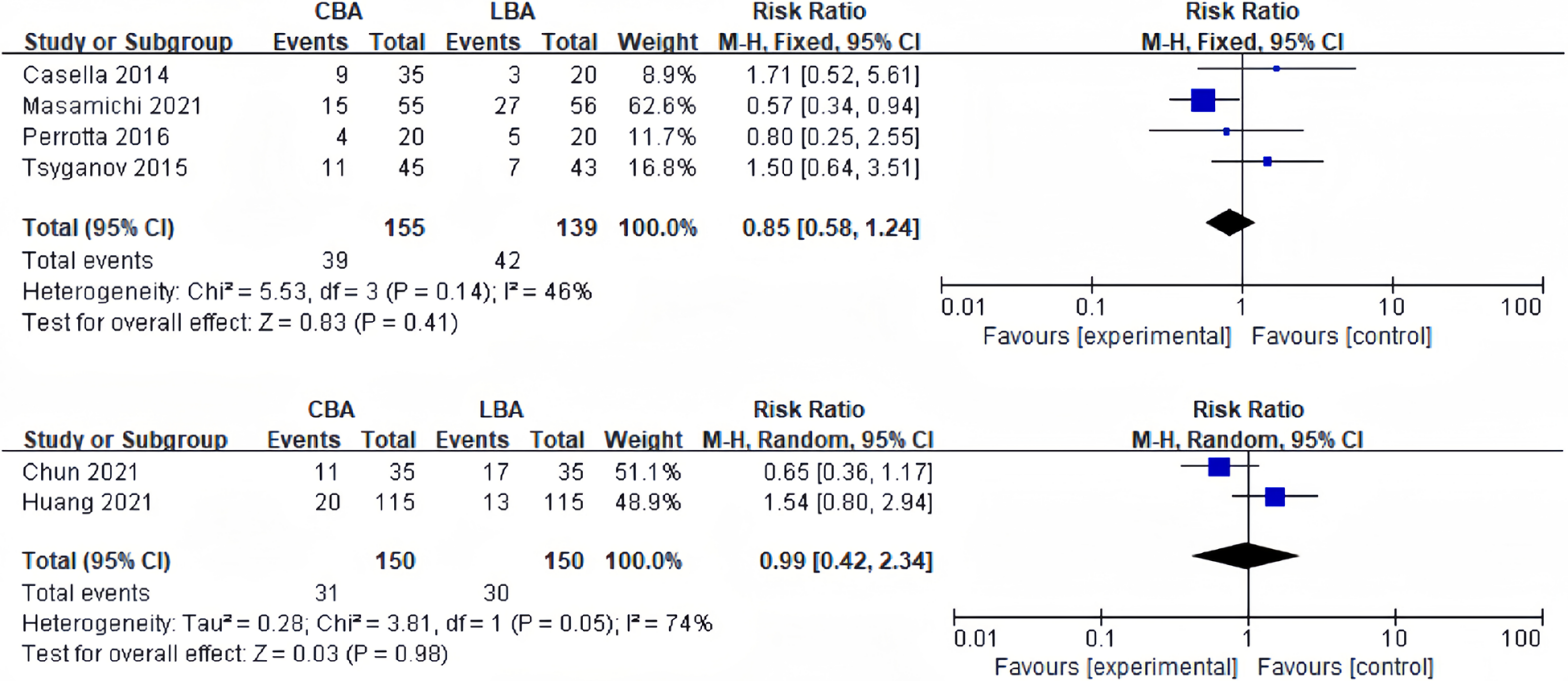
(**A**) Forest plot demonstrating 12-months recurrence rate of AF in CBA compared to LBA; (**B**) forest lot demonstrating 12-months recurrence rate of AAT in CBA compared to LBA.

4 of the 13 studies recorded 12-months recurrence rate of AF. Heterogeneity test revealed significant heterogeneity between studies (*I*^2 ^= 46%, *p* = 0.14), and a fixed effect model was applied for synthesis. The results revealed that 12-months recurrence rate of AF was 25.1% (39/155) in CBA group and 30.2% (42/139) in LBA group. The difference was not statistically significant (RR = 0.85, 95%CI: 0.58–1.24) (Figure 3A).

2 studies reported 12-months recurrence rate of atrial tachyarrhythmia (AAT). Heterogeneity test showed significant heterogeneity among the studies (*I*^2 ^= 74%, *p* = 0.05), and a random effect model was applied for synthesis. The results revealed that 12-months recurrence rate of AAT was 20.6% (31/150) in CBA group and 20.0% (30/150) in LBA group. The difference was not statistically significant (RR = 0.99, 95%CI: 0.42–2.34) (Figure 3B).

#### 3.3.3. Incidence of periprocedural complications

**Figure 4:**
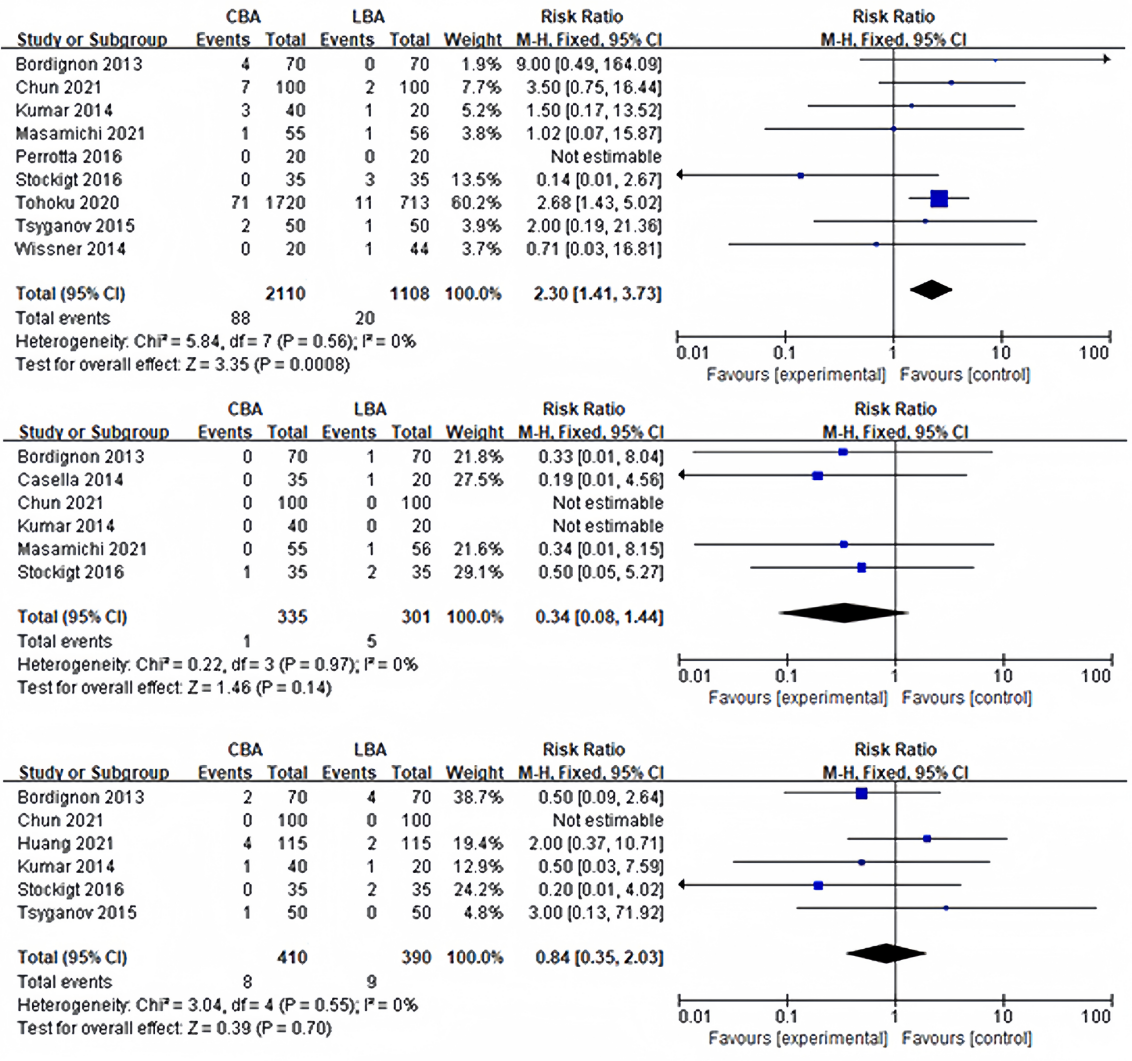
(**A**) Forest plot demonstrating incidence of postoperative transient PNP; (**B**) forest plot demonstrating incidence of postoperative tamponade; (**C**) forest plot demonstrating incidence of groin complications.

Periprocedural problems were observed in 11 of the 13 trials. Complications occurred in more than three studies were PNP, tamponade and groin complications. Other complications included bleeding (3 cases) (14) and transient ischemic attack (2 cases) (6, 10). Stroke, atrial-esophageal fistula and death were not reported. Heterogeneity test showed no significant heterogeneity between studies (transient PNP: *I*^2 ^= 39%, *p* = 0.12; persistent PNP: *I*^2 ^= 26%, *p* = 0.24; tamponade: *I*^2 ^= 0%, *p* = 0.97; groin complications: *I*^2 ^= 0%, *p* = 0.55), and a fixed effect model was used for synthesis. 9 of the 13 studies reported incidence of transient PNP. The results revealed that incidence of transient PNP in the CBA group was 2.7% (58/2,110), and that in the LBA group was 0.7% (8/1,108), and the difference was statistically significant (RR = 4.25, 95%CI: 2.06–8.76) (Figure 4A). However, the sensitivity analysis discovered that after removing the study of Tohoku (17), the difference became no longer statistically significant but still remained the trend (CBA 4.3% vs. LBA 2.3%, RR = 2.11, 95%CI: 0.93–4.78). The incidence of persistent PNP in CBA group was similar to the incidence in the LBA group (CB 1.4% vs. LB 1.0%, RR = 1.09, 95%CI: 0.55–2.14). No statistical difference was observed in the incidence of tamponade (CB 0.2% vs. LB 1.7%, RR = 0.34, 95%CI: 0.08–1.44) and groin complications (CBA 2.0% vs. LBA 2.3%, RR = 0.84, 95%CI: 0.35–2.03) (Figures 4B,C).

#### 3.3.4. Periprocedural time

**Figure 5:**
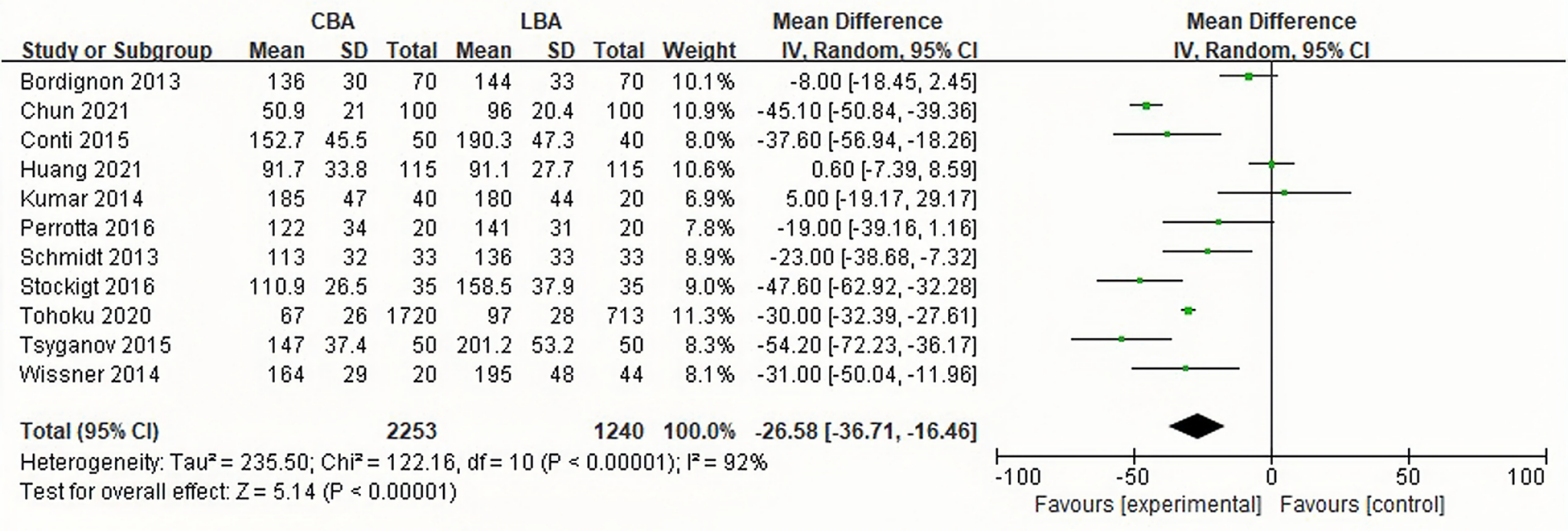
Forest plot illustrating periprocedural time.

11 of the 13 studies reported periprocedural time. The heterogeneity test showed that heterogeneity existed among the studies, and the random effect model was used for synthesis (*I*^2 ^= 92%, *p* < 0.001). The results showed that in CBA group the periprocedural time was significantly shorter than that in LBA group (WMD = −26.58, 95%CI: −36.71 to −16.46) (Figure 5).

### 3.4. Publication bias

Publication bias was assessed through funnel plots and Egger's test. Funnel plots of all the outcomes showed symmetric distribution (Supplementary Figures S2–S10). *P* value of Egger's tests were all more than 0.05, which could not indicate the existence of publication bias (Supplementary Table S2).

## 4. Discussion

In this meta-analysis, we compared the efficacy and safety of CBA and LBA in the treatment of AF. In our study, main conclusions were as follows: (1) CBA can achieve higher acute PVI rate per patient than LBA in patients with AF; (2) The incidence of transient PNP but not persistent PNP is higher in CBA group can LBA group; (3) CBA has a significantly shorter procedural time.

### 4.1. Primary efficacy outcome

The study confirmed that both CBA and LBA showed similar acute PVI rate per vein (CBA 98.7% vs. LBA 98.8%, RR = 1.00, 95%CI: 0.99–1.01). However, there was a significantly difference in acute PVI rate per patient (CBA 97.0% vs. LBA 93.4%, RR = 1.04, 95%CI: 1.01–1.07), which became insignificant (RR = 1.01, 95%CI: 0.98–1.04) after excluding the study by Stockigt (16). In the studies included, various reasons led to the failure of PVI, such as anatomic factors, increased esophageal temperature, PNP and pericardial tamponade. Several anatomic factors, including the width of the left lateral ridge and the size of right superior PV could influence the acute PVI rate or 12-months recurrence rate (22). Stockigt attributed relatively lower acute PVI rate in the LBA group to the immature technology. The acute PVI rate of LBA gradually increased as the operator became more skilled and familiar in laser balloon (16). Similar learning curve effect was observed in several studies (23, 24), in which the acute PVI failure rate, incidence of complications and procedural time decreased over time. Sensitivity analysis indicated that the difference of PVI rate in CBA and LBA groups could be partially attributed to the learning curve effect. However, in other studies included, the authors did not mention the occurrence of a learning curve effect, so it is difficult to evaluate the actual size of the effect. Subgroup analysis discovered that the acute PVI rate did not differ in CBA and LBA groups in the subgroup containing only PAF patients and subgroup containing both PAF and PersAF patients.

In terms of long-term outcomes, two ablation devices showed similar efficacy. The 12-months recurrence rates of AF after CBA and LBA pooled from 4 studies were 25.1%, and 30.2%, and the 12-months recurrence rate of AAT after CBA and LBA pooled from 2 studies were 20.6% and 20.0%. Subgroup analysis discovered that the 12-months recurrence rate did not differ in CBA and LBA groups in the subgroup containing only PAF patients and subgroup containing both PAF and PersAF patients. This is consistent with Chun's study, which discovered that 12-months success rate between CBA and LBA groups were not different in both PAF and PersAF groups (11). Relatively high 12-months recurrence rate emphasizes the utmost requirement for new technology to produce durable PVI. Contact force-guided radiofrequency ablation was proved to increase the 12-months freedom from ATA (25). Applying ablation index to CBA and LBA may be promising in improving further outcome of PVI.

### 4.2. Primary safety outcome

In terms of safety, this study suggested that CBA and LBA had similar incidence of postoperative tamponade (CBA 0.2% vs. LBA 1.7%, RR = 0.34, 95%CI: 0.08–1.44) and groin complications (CBA 2.0% vs. LBA 2.3%, RR = 0.84, 95%CI: 0.35–2.03). It is worth noting that incidence of transient PNP after CBA was significantly higher than that after LBA (CBA 2.7% vs. LBA 0.7%, RR = 4.25, 95%CI: 2.06–8.76). The difference became insignificant after excluding the study by Tohoku (17), but CBA group still presented a higher trend in the incidence of transient PNP (CB 1.4% vs. LB 1.0%, RR = 1.09, 95%CI: 0.55–2.14). This difference could be explained by a difference in ablation techniques. In CBA, the operator pushed or pulled the catheter according to x-ray image and resistance condition, which might reduce the distance the between balloon and phrenic nerve and cause damage to phrenic nerve. However, LBA could be performed under the guidance of a endoscope. It should be noted that all patients with PNI recovered during a 3-year follow-up period, but phrenic nerve damage caused by LBA lasted longer than that caused by CBA (17). 45.5% of the patients in LBA group required more than 12 months to recover, while only 7.3% of the patients in CBA group required more than 12 months. In recent YETI registry, similar results was discovered that only 3% of the patients with phrenic nerve injury induced by CBA lasted more than 12 months (26). However, it is worth noticing that damage caused by low temperature was usually more reversible (27), but damage caused by high temperature was often persistent. In our study, the incidence of persistent PNP after CBA was similar to that after LBA (CB 1.4% vs. LB 1.0%, RR = 1.09, 95%CI: 0.55–2.14), indicating that long-term safety may be similar in the two groups.

### 4.3. Secondary outcome

In this study, it was discovered that procedural time of CBA was shorter than that of LBA (WMD = −26.58, 95%CI: −36.71 to −16.46) This could be explained by the following reasons. Firstly, the temperature of balloon wall decreases synchronously and causes annular lesion, while laser balloon releases energy segmentally, generating lesion within a range of 30° each time (28), which may prolong procedural time. Secondly, real-time monitoring of pulmonary vein potential through mapping catheter was widely applied in CBA. This enables operators to develop individualized ablation strategies and save procedural time. Nevertheless, the first-generation laser balloon cannot detect real-time potential of pulmonary vein and can only be mapped after ablation. If pulmonary vein isolation is not achieved, another round of ablation is required, which undoubtedly increases the procedural time. Meanwhile, Stock mentioned that LBA procedures were interrupted more frequently due to the result of a rising esophageal temperature (16). Thirdly, learning curve effect may play a significant role. Atsushi compared procedural time of CBA and LBA during the introduction periods in the first 50 patients, and discovered that the procedural time of CBA was significantly shorter than that of LBA in the first 30 patients, but this difference disappeared after that (24). Stockigt also discovered that in the 4th quartile of all consecutive LBA patients, the procedure time was as low as that in the CBA group (16).It is worth noting that the emergence of the third-generation laser balloon greatly reduces the procedural time (29). Huang used the third-generation laser balloon for ablation, and the results showed that there was no significant difference in the procedural time between the third-generation LBA and CBA (13).

### 4.4. Strengths and limitations

Yue conducted a meta-analysis on the efficacy and safety of CBA and LBA before our study (30). Compared to the previous study, our study included a larger sample size and more randomized controlled studies, including the first large prospective randomized controlled study in this field (11). The larger sample size reduced possibility of bias. Meanwhile, Yue did not take blanking period into consideration in the calculation of 12-month recurrence. Different definition of recurrence during blanking period in included studies might lead to bias. In our study, we only included studies that did not consider recurrence during blanking period as a true recurrence.

There were also a few limitations in our study. First of all, two of the included studies were only abstracts of the conference papers, and the methods in these studies could not be fully understood, which might lead to the occurrence of bias. Second, laser balloon was a new technology, so the total sample size was still small. Third, in studies included both PAF and PersAF patients, no study reported the outcome of PAF and PersAF patients separately. All the studies reported a undistinguished result. Fourth, most of the included studies used first-generation and second-generation laser balloons, which could influence the results of the analysis. According to Tohoku, the third-generation laser balloon presented higher PVI rate than the second-generation laser balloon in AF patients (31). Fifth, Stockigt reported the learning curve effect (16), and this effect might also exist in other studies. Sixth, all the included studies were from English journals, and all the patients were Caucasian, which might lead to bias. Moreover, the ablation devices within groups are different, which causes noticeable heterogeneity.

## 5. Conclusion

Cryoballoon ablation presents a higher acute pulmonary vein isolation rate and a shorter procedural time in patients with atrial fibrillation than laser balloon ablation. There is a higher incidence of transient but not persistent phrenic nerve palsy after cryoballoon ablation. In the future, more large prospective controlled clinical studies are needed to explore the efficacy and safety differences between cryoballoon ablation and laser balloon ablation.

## Data Availability

The original contributions presented in the study are included in the article/**Supplementary Material**, further inquiries can be directed to the corresponding author.
