# Supplementary material for: Cryoballoon vs. laser balloon ablation for atrial fibrillation: a meta-analysis
Source: Front Cardiovasc Med. 2023 Dec 18;10:1278635. doi: 10.3389/fcvm.2023.1278635 (PMC10761002; doi:10.3389/fcvm.2023.1278635)
Supplement: Supplementary file 1 [file Table1.docx]

**Cryoballoon versus laser balloon ablation for atrial fibrillation: a meta-analysis**

Xiaochi Sun^△,1^, Shenyu Zhao^1^, Simin Yu^2^, Kaijun Cui^*,1^

^1^Department of Cardiology, West China Hospital, Sichuan University, No. 37, Guoxue Alley, Chengdu, Sichuan 610041, People’s Republic of China

^2^West China Medical School, Sichuan University, No. 37, Guoxue Alley, Chengdu, Sichuan 610041, People’s Republic of China

^△^Author to whom proofs should be sent. Address: West China Hospital, Sichuan University, No. 37, Guoxue Alley, Chengdu, Sichuan 610041; Tel: +86 18208143070; Fax numbers: +86 28 85422344; E-mail: 615350213@qq.com

^*^Corresponding author. Tel: +86 28 85422602; Fax: +86 28 85422344; E-mail:cuikaijun@hotmail.com

|  | P value of Egger’s test |
| --- | --- |
| Acute PVI failure per vein | 0.4743 |
| Acute PVI failure per patient | 0.9388 |
| Recurrence of AF | 0.1301 |
| Recurrence of AAT | NR^※^ |
| Phrenic nerve palsy | 0.2199 |
| Tamponade | 0.8711 |
| Groin complications | 0.8350 |
| Procedure time | 0.6828 |
| Fluoroscopy time | 0.9753 |

Table S2 P value of Egger’s test of all primary and secondary outcomes

※Only 2 studies reporting recurrence of AAT were included, which was insufficient for conducting an Egger’s test.


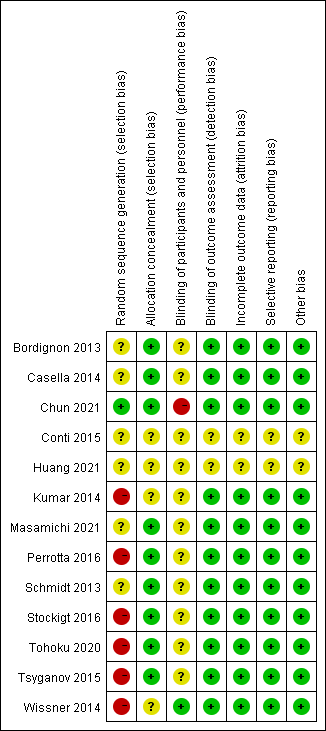


Figure S1 Risk of bias summary


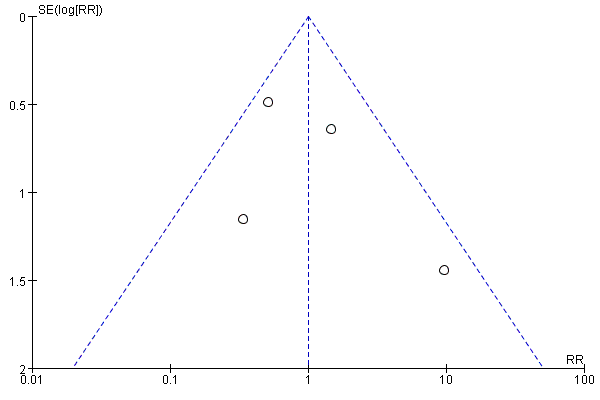


Figure S2 Funnel plot of acute PVI failure per vein


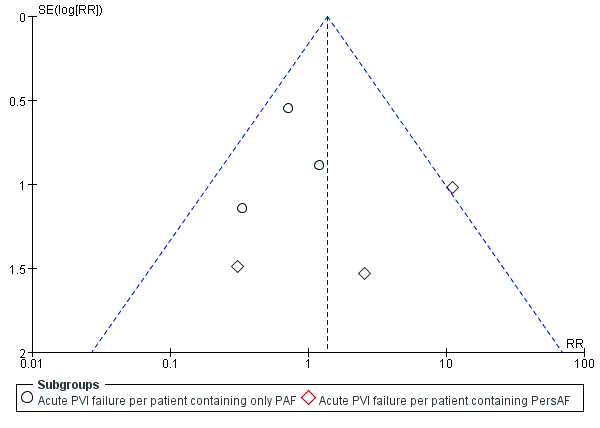


Figure S3 Funnel plot of acute PVI failure per patient


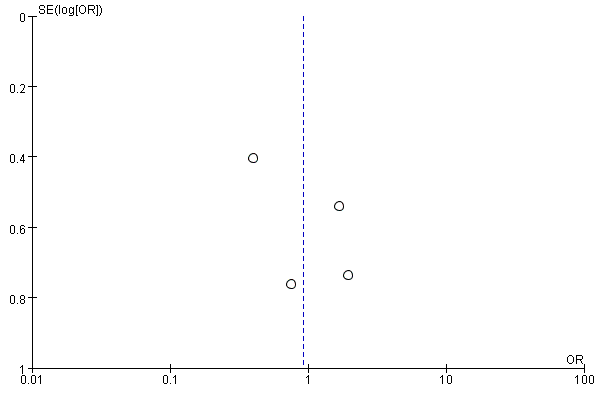


Figure S4 Funnel plot of recurrence of AF


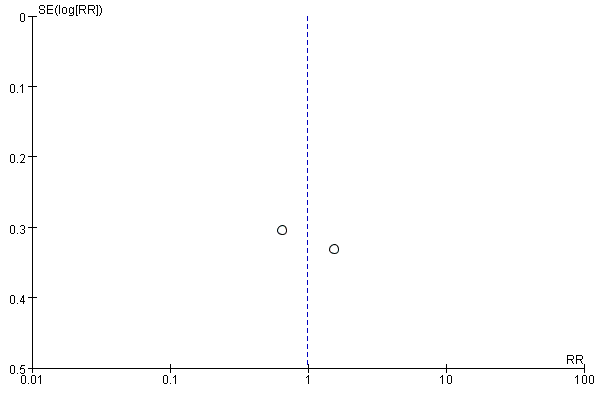


Figure S5 Funnel plot of recurrence of AAT


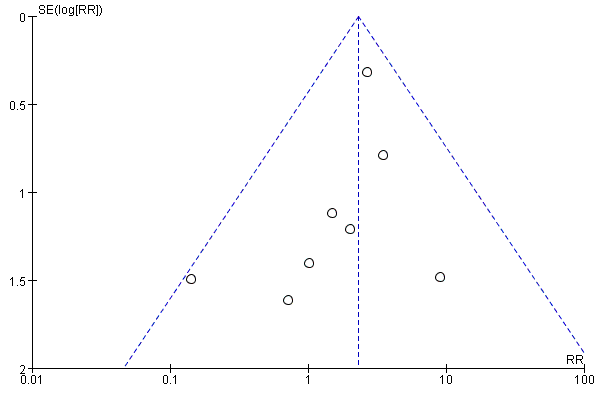


Figure S6 Funnel plot of phrenic nerve palsy


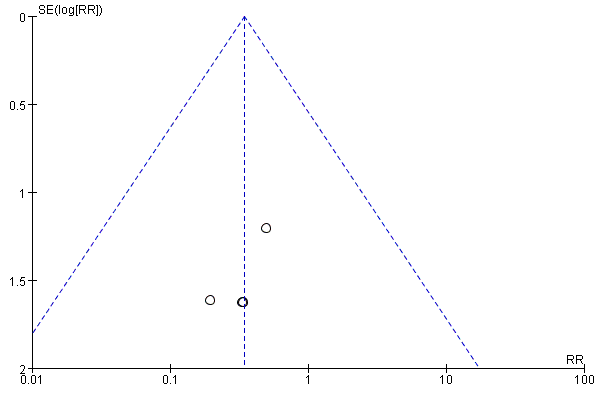


Figure S7 Funnel plot of tamponade


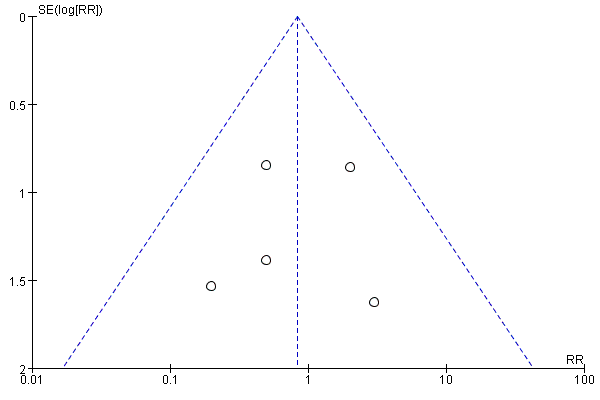


Figure S8 Funnel plot of groin complications


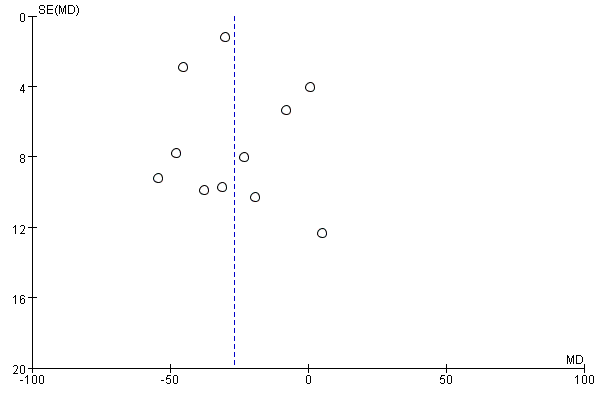


Figure S9 Funnel plot of procedure time


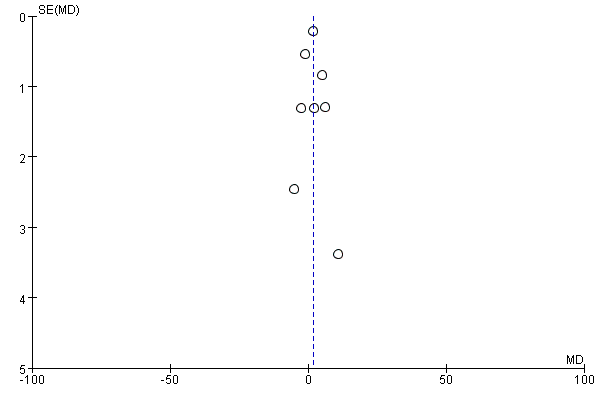


Figure S10 Funnel plot of fluoroscopy time
