# Supplementary material for: Cryoballoon vs. laser balloon ablation for atrial fibrillation: a meta-analysis
Source: Front Cardiovasc Med. 2023 Dec 18;10:1278635. doi: 10.3389/fcvm.2023.1278635 (PMC10761002; doi:10.3389/fcvm.2023.1278635)
Supplement: Supplementary file 2 [file Table2.docx]

**Cryoballoon versus laser balloon ablation for atrial fibrillation: a meta-analysis**

Xiaochi Sun^△,1^, Shenyu Zhao^1^, Simin Yu^2^, Kaijun Cui^*,1^

^1^Department of Cardiology, West China Hospital, Sichuan University, No. 37, Guoxue Alley, Chengdu, Sichuan 610041, People’s Republic of China

^2^West China Medical School, Sichuan University, No. 37, Guoxue Alley, Chengdu, Sichuan 610041, People’s Republic of China

^△^Author to whom proofs should be sent. Address: West China Hospital, Sichuan University, No. 37, Guoxue Alley, Chengdu, Sichuan 610041; Tel: +86 18208143070; Fax numbers: +86 28 85422344; E-mail: 615350213@qq.com

^*^Corresponding author. Tel: +86 28 85422602; Fax: +86 28 85422344; E-mail:cuikaijun@hotmail.com

| Author(publish year) | Inclusion criteria | Exclusion criteria | Coagulant use | Management during blanking period | Primary end point | Secondary endpoint | LB Ablation | CB Ablation |
| --- | --- | --- | --- | --- | --- | --- | --- | --- |
| Bordignon 2013 | Paroxysmal AF refractory to AAD | <18 years old or >75 years old; LA> 50mm or LAEF <45% | INR :2-3 | All previously ineffective AAD were stopped immediately after the procedure. A blanking period of 90 days was applied. | Recurrence of AF between 90 and 365 days and incidence of periprocedural complications | Procedure and fluoroscopy times and the number of acutely isolated PVs | First-generation laser balloon | Second-generation28 mm cryoballoon |
| Casella 2014 | Paroxysmal AF refractory to AAD | Renal failure, myopathy, recent myocardial ischemia  or clinical signs of infection | Activated clotting time: 300-400 s | NR | Elevation of acute myocardial injury marker and inflammation markers after ablation | Freedom from AF within 1 year; incidence of periprocedural complications | First-generation laser balloon | First-generation 23-mm or 28-mm balloon (Arctic FrontTM , Medtronic) and second-generation ion cryoballoon |
| Chun 2021 | Paroxysmal (<7 days) or persistent (>7 days and <1 year) AF refractory to AAD | <18 years old or >80 years old;  LA >45mm; LAEF <45%; having been undergone PVI before; presence of intracardiac thrombus or moderate mitral valve disease. | INR :2-3 | Use of AAD was confined to the 90 days blanking period. | Freedom form ATa within 12 months | Acute PVI achievement rate; 12 months success rate; Complications | First-generation and second-generation laser balloon (first n=32 and second generation n=68) | Second-generation CB (SC, Achieve, 20 mm, Medtronic, Minneapolis,MN) |
| Conti 2015 | Paroxysmal AF | NR | NR | NR | Rate of complete isolation of all PVs and AF-free survival after 12 months | Procedure and fluoroscopy durations and occurrence of complications | First-generation laser balloon | Second-generation 28-mm CB (Arctic Front Advance, Medtronic Inc.) |
| Huang 2021 | Paroxysmal AF | NR | NR | NR | Freedom form ATa within 12 months | Procedural parameters and procedural complications. | Third-generation Laserballoon (Heartlight X3; LB3) | Third-generation cryoballoon(Arctic Front Advance-Short Tip; CB3) |
| Kumar 2016 | Paroxysmal and persistent AF refractory to AAD | Prior left atril procedures, LA diameter >50mm, obstructive pulmonary disease, left ventricular ejection fraction <30 %, presence of intracardiac thrombus, moderate or severe valvular heart disease, myocardial infarction or cardiac surgery within the prior 3 months, and stroke or transient ischemic attack in the prior 6 months | NR | AADs were stopped 3 months after the procedure, only if there was no arrhythmia during follow-up. | PV reconduction after ablation using adenosine testing | Freedom from AF within 1 year | Endoscopic ablation system(HeartLight™, CardioFocus Inc. LB, CardioFocus Inc., Marlborough, MA, USA) | Second-generation CB (Arctic Front™, Medtronic; CB, Medtronic, Minneapolis, MN, USA) |
| Masamichi 2021 | Paroxysmal AF refractory to AAD | NR | Anticoagulation therapy was started at least 3 weeks before the ablation procedure. | NR | Elevation of acute myocardial injury marker and inflammation markers after ablation | Freedom from AF or atrial tacycardia within 1 year | First-generation laser balloon | Second-generation CB over an inner-lumen circumferential mapping catheter (Achieve™, Medtronic). |
| Perrotta 2016 | Paroxysmal and persistent AF refractory to AAD | <18 years old or >80 years old; LA diameter >55mm; valvular dysfunction >II; or a LVA prior to PVI detected by bipolar voltage mapping | Activated clotting time: 300-350 s | NR | Size of the isolated surface area acutely after PVI | Freedom from AF within 1 year; incidence of periprocedural complications | Visually guided laser ablation catheter | Second-generation 28-mm balloon  (Arctic Front AdvanceTM, Medtronic) |
| Schimidt 2013 | Paroxysmal AF refractory to AAD | LA> 50mm; LAEF <45%; any contraindications for MRI scanning; stage III renal failure; presence of an intracardiac thrombus or a CHADS score >3 | INR :2-3 | NR | Incidence of asymptomatic cerebral lesions | Acute rate of PVI, freedom from AF within 1 year and procedural characteristics | Visually guided laser ablation catheter | First-generation 28-mm balloon (Arctic FrontTM , Medtronic) |
| Stockigt 2016 | Persistent or longstanding AF refractory to AAD | Prior ablation procedure | INR <2.5 | A blanking period of 90 days was applied. | Rate of complete isolation of all PVs and AF-free survival after 12 months | Procedure and fluoroscopy durations and occurrence of complications | Visually guided laser ablation catheter | Second-generation 28-mm CB (Arctic Front Advance, Medtronic Inc.) |
| Tohoku 2020 | Paroxysmal and persistent AF refractory to AAD | NR | NR | NR | Incidence of phrenic nerve palsy | NR | NR | NR |
| Tsyganov 2015 | Paroxysmal AF refractory to AAD | Left ventricular dysfunction | Activated clotting time: 300-350 s | A blanking period of 90 days was applied. | Procedural achievement of proven electrical isolation of all veins | Freedom from AF within 1 year | Visually guided laser ablation catheter (HeartLightTM, CardioFocus, MA, US) | Second generation cryoballoon(Arctic Front AdvanceTM, Medtronic, MN, US) |
| Wissner 2014 | Paroxysmal and short-standing persistent AF refractory to AAD | A previous left atrial (LA) ablation procedure,long-standing persistent AF, LA diameter >60 mm, severe valvular heart disease, or contraindications to post-interventional oral anticoagulation. | INR :2-3 | Previously ineffective antiarrhythmic drug therapy was continued for 3 months. | Incidence of new asymptomatic brain lesions | Rate of achivement of acute PVI and incidence of periprocedural complications | Visually guided laser ablation catheter | Second-generation 28-mm balloon  (Arctic Front AdvanceTM, Medtronic) |

**Table S1 Characteristics of included studies**
